# A novel cysteine desulfurase influencing organosulfur compounds in *Lentinula edodes*

**DOI:** 10.1038/srep10047

**Published:** 2015-06-09

**Authors:** Ying Liu, Xiao-Yu Lei, Lian-Fu Chen, Yin-Bing Bian, Hong Yang, Salam A. Ibrahim, Wen Huang

**Affiliations:** 1 College of Food Science and Technology, Huazhong Agricultural University, Wuhan, Hubei, 430070, China; 2 Institute of Applied Mycology, College of Plant Science and Technology, Huazhong Agricultural University, Wuhan, Hubei, 430070, China; 3 Department of Family and Consumer Sciences, North Carolina A&T State University, 171 Carver Hall, Greensboro, NC 27411, United States

## Abstract

Organosulfur compounds are the basis for the unique aroma of *Lentinula edodes*, and cysteine sulfoxide lyase (C-S lyase) is the key enzyme in this trait. The enzyme from *Alliium sativum* has been crystallized and well-characterized; however, there have been no reports of the characterization of fungi C-S lyase at the molecular level. We identified a *L. edodes* C-S lyase (Lecsl), cloned a gene of *Csl* encoded Lecsl and then combined modeling, simulations, and experiments to understand the molecular basis of the function of Lecsl. Our analysis revealed Lecsl to be a novel cysteine desulfurase and not a type of cysteine sulfoxide lyase. The pyridoxal-5-phosphate (PLP) molecule bonded tightly to Lecsl to form a Lecsl-PLP complex. Moreover, the Lecsl had one active center that served to bind two kinds of substrates, S-methyl-L-cysteine sulfoxide and L-cysteine, and had both cysteine sulfoxide lyase and cysteine desulfurase activity. We found that the amino acid residue Asn393 was essential for the catalytic activity of Lecsl and that the gene *Csl* encoded a novel cysteine desulfurase to influence organosulfur compounds in *L. edodes*. Our results provide a new insight into understanding the formation of the unique aroma of *L. edodes*.

*lentinula edodes* (shiitake mushroom) is rich in sulfur and is known for its nutritional value as well as its application in traditional medicine. Sulfur plays a major role in biology and is found in numerous peptides, proteins and low molecular weight compounds^1^,^2^. Lenthionine (1,2,3,5,6-pentathiepane), a cyclic sulfur compound found in *L. edodes*, is the basis for the unique aroma of the shiitake mushroom^3^,^4^. In addition, lenthionine has been found to have antibiotic properties against bacteria and fungi^5^,^6^,^7^, and show measurable inhibitory activity against platelet aggregation^8^. Lenthionine is derived from a γ-L-glutamyl-cysteine sulfoxide precursor (lentinic acid) in a two-step enzymatic reaction^9^,^10^. Lentinic acid is first activated by the removal of its γ-glutamyl moiety catalyzed by γ-glutamyl transpeptidase (GGT) producing a L-cysteine sulfoxide derivative **1** which then undergoes α, β-elimination catalyzed by cysteine sulfoxide lyase (often called alliinase in garlic and onion), resulting in a highly reactive sulfenic acid **2** intermediate^11^. The sulfenic acid is then rapidly condensed to form thiosulfinate **4** (Fig. 1), and the thiosulfinate is often further transformed into other sulfur compounds including lenthionine.

Cysteine sulfoxide lyase (EC 4.4.1.4, also named alliinase) is a pyridoxal-5-phosphate (PLP) dependent enzyme and belonging to the class I family of PLP dependent enzymes^12^. Alliinase is well characterized in *A. Sativum* and is a homodimeric glycoprotein in which each subunit consists of 448 amino acids accounting for a molecular weight of 51,500 Da^13^,^14^. *Allium* alliinases are also purified and characterized from *Allium cepa*, *Allium porrum*, *Allium tuberosum Allium subgenus*, and *Allium tripedale*^15^,^16^,^17^,^18^,^19^. The affinity of the enzyme for substrate, expressed as *K*_m_ using S-ethyl-L-cysteine sulfoxide as the substrate, varies depending on the alliinase source, ranging from 2.7 mM for *Allium tuberosum*^17^ to 16 mM for *Allium cepa*^20^. Genes encoding alliinase are isolated from some *Allium* genus plants, and the sequence homology varies from 51% to 70%^21^. The C-S lyase form *L. edodes* (named Lecsl) was first described in 1971^10^. It is a PLP-dependent enzyme and has broad substrate specificity compared to *Allium* alliinases^22^. The N-terminal amino acid sequence of Lecsl is different from that of *A. sativum* alliinase^23^. There has been no identified homology sequences with *Allium* alliinases in the *L. edodes* genome. As a result, it is not known whether there is an enzyme different from other alliinases that exists in *L. edodes* and which also has cysteine sulfoxide lyase activity.

In the present study, we attempted to resolve these questions through a combination of proteomic, computational and biochemical approaches. The gene of *Csl* encoded Lecsl was first identified, cloned and sequenced. Our molecular docking results and enzyme activity assay demonstrated that Lecsl had both cysteine sulfoxide lyase and cysteine desulfurase activity. Finally, we found that Lecsl influenced the formation of organosulfur compounds in *L. edodes*.

## Results and Discussion

### Identification of a putative C-S lyase gene

**Figure 2:**
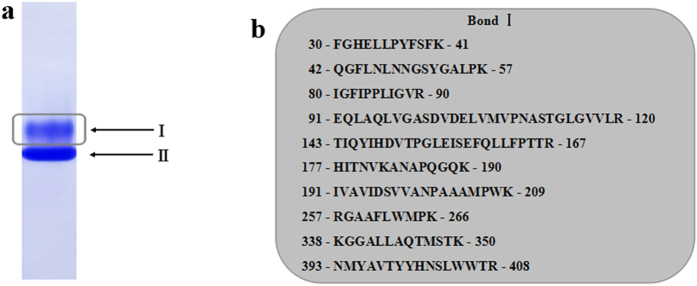
Identification of a *L. edodes* cysteine sulfoxide lyase (Lecsl). (**a**) The purified cysteine sulfoxide lyase was analyzed by Native-PAGE, and the protein bands were visualized by staining with Coomassie Blue. (**b**) The protein band I which showed cysteine sulfoxide lyase activity was subjected to protein identification by MS/MS. Among the tryptic peptides obtained from the protein band, 10 peptides matched to a putative cysteine desulfurase protein from *L. edodes.*

The purification of C-S lyase from *L. edodes* is not novel^22^,^23^,^24^,^25^,^26^, while the identification of a *L. edodes* gene that encodes C-S lyase is. The purified Lecsl was identified in two protein bands (band I and band II) in Native-PAGE (Fig. 2a). We found the band I showed cysteine sulfoxide lyase activity, while band II showed no cysteine sulfoxide lyase activity. Band I and band II were then analyzed by NanoLC-MS/MS peptide sequencing. To increase our confidence in detectable peptides, we analyzed the sample three times. Numerous peptides of the protein in band I were sequenced (Fig. 2b) and matched precisely with one protein deduced from the scaffold 356.7 in the draft genome of *L. edodes*. After a search of *L. edodes* genome, it was found that the scaffold 356.7 was a 3314 bp genomic DNA fragment encoding the putative Lecsl gene named *Csl*. The gene *Csl* encompassed the entire cDNA in 9 putative exons (see Supplementary Fig. S1a online). A consensus cDNA sequence was assembled from the sequenced clone and genomic sequence reads and was found to encode a 497-aa polypeptide (54 kDa) with a theoretical pI 6.13. A Signal P analysis by SignalP 4.0 indicated no signal peptide at the N-terminus of Lecsl. The predicted amino acids sequence of Lecsl was then analyzed by InterProScan, and a region (amino acids 86—367) was identified with homology to aminotransferase, class V/cysteine desulfurase (EC 2.8.1.7) (see Supplementary Fig. S1b online). Numerous peptides of the protein in band II were also sequenced (see Supplementary Fig. S2 online) and identified as a putative phosphatidylserine decarboxylase (EC 4.1.1.65) protein from *L. edodes.* The molecular weight of phosphatidylserine decarboxylase (54 kDa) was the same as Lecsl. Although it is typically difficult to separate two proteins with the same molecular weight by SDS-PAGE for peptide sequencing, we were able to successfully accomplish this task. We thus propose that Native-PAGE is an effective method of purifying a bioactive protein for peptide sequencing.

### Sequence analysis of Lecsl

A search for homologous sequences in NCBI protein database revealed that Lecsl shared a strong similarity with a group of known or putative cysteine desulfurases from archaea, bacteria, fungi, animals, and plants. The highest degrees of homology were with sequences from fungi. However, there was no significant homology between the predicted Lecsl sequence and other available cysteine sulfoxide lyase sequences. It is notable that Lecsl shares no homology with cysteine sulfoxide lyases. The cysteine sulfoxide lyase has already been crystallized and its three-dimensional structure solved^12^,^21^. We found that the predicted Lecsl sequence shows no conservation of the active residues compared with the structured cysteine sulfoxide lyases (PDB: 1L9K, 2HOX, 2HOR) sequences. The cDNA of Lecsl gene does not, by itself, seem to be sufficient to support cysteine sulfoxide lyase activity. Our results thus suggest that Lecsl is a novel cysteine desulfurase.

**Figure 3:**
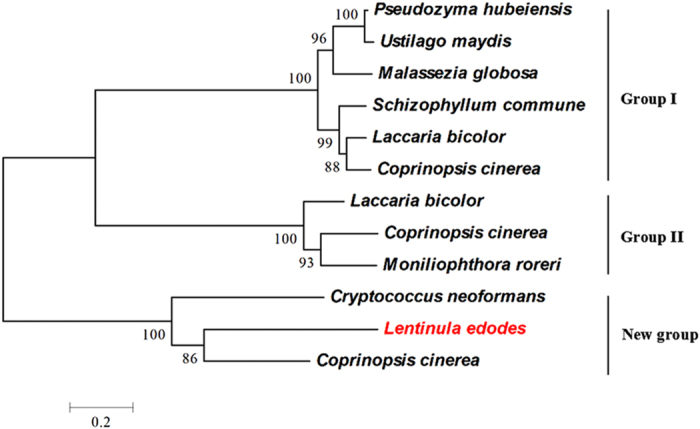
Phylogenetic tree of Lecsl (red) and related cysteine desulfurases. The tree was constructed using the MEGA 4.1 program with the neighbor-joining method. Bootstrap values are indicated at branchpoints. Sequences were obtained from Genbank and sequenced genomes.

Cysteine desulfurase removes persulfide from cysteine to provide it for various recipients such as Fe-S clusters^27^. Cysteine desulfurase also shows selenocysteine lyase activity *in vitro* by cleaving selenocysteine to alanine and elemental selenium^28^. All organisms studied to date encode cysteine desulfurases which are termed NifS/IscS (group I), CsdA/SufS (group II) in bacteria and Nfs in mitochondria^29^. To clarify the possible evolutionary position of Lecsl and its homologs within a range of *Basidiomycetes*, a phylogenetic tree was constructed based on the classification of bacterial cysteine desulfurases^30^,^31^. Analysis of the phylogenetic tree showed that Lecsl was not classified into any of the two groups and instead formed its own distinctive group (Fig. 3). The enzymes belonging to this new group have not been identified or characterized to date. In addition, various cysteine desulfurases from *Coprinopsis cinerea*, and *Laccaria bicolor* were found in the different groups. The results suggest that there are additional cysteine desulfurases, not only Lecsl, in *L. edodes*.

It is well known that both cysteine desulfurase and cysteine sulfoxide lyase are PLP-dependent enzymes^12^,^30^. In addition, these enzymes belong to the fold type I, a group that contains aspartate aminotransferase as its most representative member^21^,^32^,^33^,^34^,^35^. The PLP-dependent enzymes are not only involved in the biosynthesis of amino acids and amino acid-derived metabolites, but also play key roles in the replenishment of one-carbon units, assimilation and metabolic transformations of nitrogen and sulfur-containing compounds, synthesis of tetrapyrrolic compounds and metabolism of amino-sugars^36^,^37^,^38^. The consequence of these enzymes’ crucial metabolic relevance is that a number of them are widely recognized drug targets^28^,^39^. We have identified a gene of *Csl* that encodes a novel cysteine desulfurase in *L. edodes.* To our knowledge, this is the first time that a cysteine desulfurase gene has encoded a protein with cysteine desulfurase activity.

In order to investigate the conserved catalytic residues of Lecsl, amino acid sequence alignments with other structurally characterized cysteine desulfurases and cysteine sulfoxide lyases were performed using ClustalW2. The results indicated that Lecsl covered similar cysteine desulfurase conservative amino acid residues with known functions (see Supplementary Fig. S3 online). According to the locations of these residues, it was assumed that the amino acid residues Ser112, Asp225, His228, Asn248, His250 and Lys251 (Schiff-base linkage) were tightly bonded to the PLP, and the amino acid residues Lys388, Asn393 and Arg408 were possibly involved in the catalytic reaction of cysteine desulfurase^40^,^41^,^42^,^43^. The results indicate that Lecsl may thus have cysteine desulfurase activity. We also found that there were some striking features, such as the replacement of Thr100/95/91, Gln208/203/199, Ser228/223/219, Arg367/359/353 and Cys372/364/358 in SynSufs/EcSufs/EcCsdA by Ser112, His228, Asn248, Lys388 and Asn393 in Lecsl. It has been reported that Cys372/364/358 is essential for the catalytic activity of SynSufs/EcSufs/EcCsdA, which reacts to attack of the nucleophilic Cys on the substrate sulfur^41^,^42^,^43^. However, the Cys residue is replaced by Asn393 in Lecsl, which may be necessary for the catalytic activity of Lecsl. As a result, we computed the structure of Lecsl to investage its catalytic function.

### Molecular modeling

**Figure 4:**
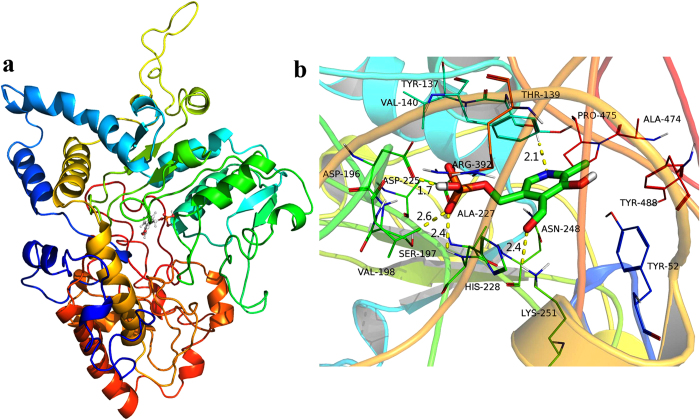
The binding model of Lecsl-PLP complex was shown by PyMoL (http://www. pymol.org/). Overview of the average coordinates of PLP in complex (**a**). Detailed view of the H-bond network on the binding region (**b**), the carbon atoms of PLP were coloured in green, the nitrogen atom of PLP was coloured in blue, the oxygen atoms of PLP were coloured in red and the phosphorus atom of PLP was coloured in orange.

The cofactor PLP is essential for the function of PLP-dependent enzymes. To predict the binding mode between the novel cysteine desulfurase Lecsl and PLP, a docking study of PLP was carried out using AutoDock Vina, and MD simulations were then performed to refine the docking result by GROMACS. The 3D structure of Lecsl was performed by the I-Tasser server. The Lecsl-PLP complex (Fig. 4a) was equilibrated after 40 ns MD simulation, and the plot of RMSD (in ångstrom) of the complex is shown as Supplementary Fig. S4 online. As shown in Fig. 4b, the pyridine ring of PLP adopted a compact conformation to bind inside the hydrophobic pocket formed by the amino acid residues Pro475, Ala474, Tyr488 and Tyr52 (Fig. 4b). The PLP pyridine ring nitrogen atom was involved in hydrogen- bonding interactions with the hydroxyl hydrogen atom of Thr139 (the distance is 2.1 Å). Importantly, our model showed that the previously proposed role of the conserved Asp225, His228, Asn248 and Lys251 in binding its cofactor PLP. Accordingly, the aldehyde group oxygen atom of PLP formed a hydrogen bond with the ε-amino group hydrogen atom of Lys251 (the distance is 2.4 Å). It was also reported that PLP bonded through an aldimine linkage to the ε-amino group of a Lys residue located at the PLP-dependent enzyme active site^33^. The phosphoryl groups oxygen atom of PLP formed a hydrogen bond with the amidogen hydrogen atom of His228 (the distance is 2.4 Å). In addition, the Asp225 and Asn248 made a high density of vander Waals contacts to PLP. In particular, our model showed that the phosphoryl groups of PLP were also involved in hydrogen-bonding interactions with Asp196 (the distance is 1.7 Å) and Ser197 (the distance is 2.6 Å). Overall, the PLP molecule is held very tightly by the enzyme to form a Lecsl-PLP complex, which may reflect the need to retain the cofactor during the catalytic reaction.

**Figure 5:**
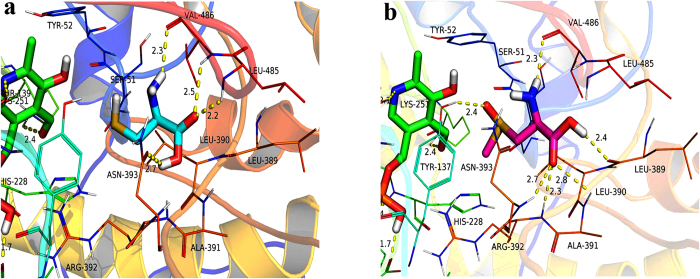
The binding models of Lecsl-PLP-L-cysteine complex (**a**) and Lecsl-PLP-S- methyl-L-cysteine sulfoxide complex (**b**) were shown by PyMoL (http://www. pymol.org/). The carbon atoms of L-cysteine were coloured in sky blue, the nitrogen atom of L-cysteine was coloured in blue, the oxygen atoms of L-cysteine were coloured in red and the sulphur atom of L-cysteine was coloured in yellow. The carbon atoms of S-methyl-L-cysteine sulfoxide were coloured in pink, the nitrogen atom of S-methyl-L-cysteine sulfoxide was coloured in blue, the oxygen atoms of S-methyl-L-cysteine sulfoxide were coloured in red and the sulphur atom of S-methyl-L-cysteine sulfoxide was coloured in yellow.

In the present study, we have proposed that Lecsl is a novel cysteine desulfurase. To predict its substrate binding mode, a docking study of two kinds of substrates L-cysteine and S-methyl-L-cysteine sulfoxide molecules was performed using AutoDock Vina. As expected from the close similarity of the chemical structures of the two molecules, docking results showed that L-cysteine and S-methyl-L-cysteine sulfoxide complex adopted a similar binding mode on Lecsl-PLP (Fig. 5). Indeed, for both ligands, the amidogen groups were involved in hydrogen-bonding interactions with the carboxylic group of Val486 (the same distance is 2.3 Å), and the carbonyl groups were inserted in a small hydrophobic pocket formed by the Leu389, Leu390, Ala391 and Leu485. However, various binding modes of the two substrates were found. The carbonyl group of L-cysteine was involved in hydrogen-bonding interactions with Leu485, Val486 and Asn393 (Fig. 5a). For S-methyl-L-cysteine sulfoxide, its carbonyl group was involved in hydrogen-bonding interactions with Ala391, Arg392, Asn393 and Leu389 (Fig. 5b). In contrast, the sulfoxide group of S-methyl-L-cysteine sulfoxide group formed a hydrogen bond with the benzene hydroxyl of Tyr137. The results indicated that Lecsl had one active center that bonded S-methyl-L-cysteine sulfoxide and L-cysteine.

We have suggested that the amino acid residue Asn393 of Lecsl is necessary for Lecsl’s catalytic activity. Based on the docking results, we defined Asn393 as being essential for Lecsl to bind the two kinds of substrates. It has been reported that the carbonyl group of substrate S-allyl-L-cysteine sulfoxide bonded tightly to the Arg401 of cysteine sulfoxide lyase, which was essential for lyase activity^12^,^21^. We also found that the carbonyl group of substrate S-methyl-L-cysteine sulfoxide bonded to the Arg392 of Lecsl, which may be necessary for Lecsl’s cysteine sulfoxide lyase activity. As a result, further studies on the function of active sites Asn393 and Arg392 should be undertaken to validate this. Our results suggest that Lecsl has both cysteine sulfoxide lyase and cysteine desulfurase activity.

### Expression and characterization of recombinant Lecsl

Based on computational insights, we carried out biochemical tests, including protein expression, purification, and enzyme activity assays. A range of bacterial expression vectors, empty or containing Lecsl, were prepared to examine the in vitro activity of the recombinant protein products. Recombinant Lecsl was synthesized in *E. coli* and purified using His tag purification. We found that the purified recombinant Lecsl showed evidence of both cysteine sulfoxide lyase and cysteine desulfurase activity.

The kinetic constants of recombinant Lecsl were then pursued in oder to uncover the possible relationship between its cysteine sulfoxide lyase and cysteine desulfurase activity. Replicate assays for cysteine sulfoxide lyase activity were conducted at varying concentrations of S-methyl-L-cysteine sulfoxide (see Supplementary Fig. S5 online). A theoretical *K*_m_ value of 26.17 ± 1.8 mM S-methyl-L-cysteine sulfoxide, a *k*_cat_ value of 33.95 ± 1.5 S^−1^ and a *k*_cat_/*K*_m_ value of 1297 M^−1^S^−1^ were calculated from the Lineweaver-Burk plot (see Supplementary Table S1 online). It has been reported that the *K*_m_ values of two isoforms with alliinase activity from onion roots were 47 mM and 96.6 mM using S-methyl-L-cysteine sulfoxide as a substrate^44^. This difference in *K*_m_ values may be due to detection methods and the varied sources of C-S lyases. In order to investigate the cysteine desulfurase activity of Lecsl, we analyzed its catalytic activity at varying concentrations of L-cysteine (see Supplementary Fig. S6 online). We then calculated a *K*_m_ value of 1.72 ± 0.3 mM, a *k*_cat_ value of 1.01 ± 0.2 S^−1^ and a *k*_cat_/*K*_m_ value of 586 M^−1^S^−1^ for Lecsl dependent on L-cysteine (see Supplementary Table S1 online). The *K*_m_ values of cysteine desulfurase from different organisms vary from 0.043 mM to 5.1 mM using L-cysteine as a substrate^45^,^46^. It has been reported that the values of *k*_cat_/*K*_m_ represented the catalytic efficiencies of enzymes^47^,^48^. We found that the *k*_cat_/*K*_m_ value of Lecsl using S-methyl-L-cysteine sulfoxide as a substrate was higher than that of Lecsl using L-cysteine as a substrate. It has been reported that the catalytic efficiency of Lecsl for its natural substrates is higher than that of Lecsl for S-methyl-L-cysteine sulfoxide. Our findings indicate that Lecsl mainly shows cysteine sulfoxide lyase activity *in vivo*.

### Effects of Lecsl on the generation of organosulfur compounds in *L. edodes*

To determine the function of Lecsl in the generation of organosulfur compounds, we analyzed the organosulfur compounds in *L. edodes* treated with recombinant Lecsl using HPLC-APCI-MS/MS. The organosulfur compound thiosulfinate **4** was detected in the Lecsl group compared with the control group. The formation of organosulfur compounds involves two processes, enzymic reactions of lentinic acid as substrate and nonenzymic thermal decomposition of thiosulfinate during processing^49^. Chen and Ho reported that lenthionine, 1,2,4,5-tetrathiane and 1,2,4-trithiolane were the major organosulfur compounds in the shiitake mushroom anzlyzed by GC-MS^50^. These different results may be due to the sample preparation methods and analytical techniques. It has been reported that some of organosulfur compounds from *Allium* are actually artifacts of the analytical techniques used^51^,^52^,^53^. Artifacts can arise from thermal breakdown or reaction in the injection port of a gas chromatograph (GC)^54^. The results show that Lecsl played an essential role in the formation of thiosulfinate in *L. edodes*. In contrast to garlic, the sulfoxide compounds in *L. edodes* are not likely to act as a deterrent to herbivores, and it is unknown why *L. edodes* produces such large quantities of exotic sulfur compounds^11^. Thus, further investigation into the biological role of sulfur compounds in *L. edodes* is warranted in the future. Our study provides support for a new approaches to studying the metabolism of sulfur in *L. edodes*.

In conclusion, we identified a putative protein gene from *L. edodes* and provided the first evidence that this protein has both cysteine sulfoxide lyase and cysteine desulfurase activity with a possible role in sulfur metabolism. Although the biological purpose of Lecsl remains to be elucidated, their highly conserved presence is presumably important for an essential function that is specific to *L. edodes*. Lecsl was probably the first example of a fungal cysteine desulfurase and thus belongs to a different protein family than other cysteine sulfoxide lyases. Our results support an enhanced understanding of a significantly large class of PLP-dependent enzymes.

## Methods

### Identification of Lecsl

Lecsl was purified from *L. edodes* following a previously described protocol^26^. Then, the purified Lecsl protein was analyzed by Native-PAGE and the protein band which had cysteine sulfoxide lyase activity was digested in the gel with sequencing-grade trypsin and subjected to peptide sequencing by tandem mass spectrometry (MS/MS). An ion trap mass spectrometer LCQ coupled with a high-performance liquid chromatography (HPLC) system running a 75-μM inner diameter C18 column was used. The collected mass spectrometric data were used in a search for the most recent non-redundant protein database (NR database, NCBI) and *L. edodes* protein database (predicted protein using *L. edodes* genome) with the ProtQuest software suite provided by ProtTech Inc. (Norristown, PA).

### Gene cloning

Total RNA extracted from *L. edodes* was used as a template for cDNA synthesis using random hexamers. The cDNA (1494 bp) was PCR amplified using Taq polymerase (Takara) and the following primers: 5`-CTAGCTAGCATGTCCAATACACAATCCATCGCGC-3` and 5`-CCGCTCGAGTCAAGGCCTAATGGAAGCAAGCTGC-3`. The resulting PCR product was cloned into the PET28a vector with an N-terminal His-tag. Successfully constructed plasmids were further confirmed by DNA sequencing.

### Molecular modeling

The three-dimensional (3D) protein structure of Lecsl was performed using the I-Tasser server^55^,^56^. Then, the AutoDock Vina^57^ (http://vina.scripps.edu/) was used to dock all small molecules against Lecsl. The 3D protein structure of Lecsl was used as the receptor for docking while the PLP, L-cysteine and S-methyl-L-cysteine sulfoxide molecules were used as ligands. Prior to docking, the receptor and ligands were converted to the PDBQT format using AutoDock Tools. For Vina docking, the default parameters were used unless otherwise specified. The affinity energy value (kcal mol^−1^) for each ligand was calculated and denoted by E_VINA_. Thereafter, a molecular dynamics (MD) study was performed to revise the docking result. The GROMACS^58^ (http://www.gromacs.org/) program was used for MD simulations of the selected docked pose. The system was equilibrated for 200 ps under NVT ensemble and another 200 ps under NPT ensemble. Standard Berendsen barostat and Langevin thermostat procedures implemented in GROMACS were used. MD simulations were performed under NPT ensemble for 40 ns and the convergence criterion is the root-mean-square deviation (RMSD) of the energy gradient. All molecular dynamics were performed on a Dell Precision T5500 work station.

### Protein expression and purification

The recombinant Lecsl was expressed in *E. coli* strain BL21 (DE3). Cells were grown at 37 °C in lysogeny broth (LB) medium containing 50 μg ml^−1^ kanamycin to an OD_600_ of 0.6, and were then induced by the addition of 0.1 mM isopropyl β-D- thiogalactoside (IPTG) at 16 °C for 12 h. Cells were harvested and re-suspended in the binding buffer (25 mM Tris-HCl, pH 8.0, 300 mM NaCl), disrupted by sonication and centrifuged at 10 000  ×  g for 20 min at 4 °C. The solution protein fe of ein by 0, 300 mMwas loaded onto a nickel Hi-trap column (Amersham Pharmacia Biotech). Elution was achieved with a 20-250 mM imidazole linear gradient.

### Effects of Lecsl on the generation of organosulfur compounds in *L. edodes*

In order to investigate the effects of Lecsl on the generation of volatile organosulfur compounds in *L. edodes*, the fresh fruiting body of *L. edodes* samples were cut into small pieces and treated at 100 °C for 5 min to inactivate the enzymes. The treated samples were then divided into two groups and homogenized in 100 mM Tris-HCl buffer (pH 8.4) separately. The purified GGT from *L. edodes* was added to group 1 (control group), and both the purified GGT^26^ and recombinant Lecsl were added to group 2. Each group was then incubated at 37 °C for 1 h. After incubation, the slurry was extracted with dichloromethane. Organic layers were combined and evaporated under reduced pressure. The obtained completely dried extracts were stored at −20 °C. For measurements, extracts were solubilized with acetonitrile and filtered with a syringe filter (Waters, Milford, MA, USA ) for HPLC-APCI-MS/MS analysis. The HPLC using a Shim-pack VP-ODS column (150 mm × 4.6 mm I. dia, 5 μm, SHIMADZU, Japan) was performed to separate organosulfur compounds. MS was carried out in negative APCI mode and an optimized collision energy of −32 eV.

## Additional Information

**How to cite this article**: Liu, Y. *et al*. A novel cysteine desulfurase influencing organosulfur compounds in *Lentinula edodes*. *Sci. Rep.* **5**, 10047; doi: 10.1038/srep10047 (2015).

## Supplementary Material

Supplementary Information

## Figures and Tables

**Figure 1:**
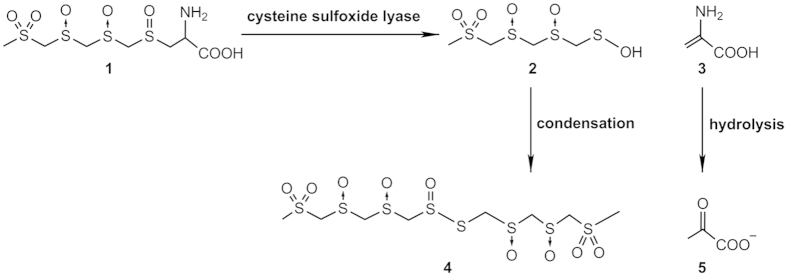
Proposed general mechanism for the catalysis of L-cysteine sulfoxide derivative from L. edodes by cysteine sulfoxide lyase. 1, L-cysteine sulfoxide derivative; 2, sulfenic acid; 3, α-aminoacrylic acid; 4, thiosulfinate; 5, pyruvate.
