# Supplementary material for: A novel cysteine desulfurase influencing organosulfur compounds in Lentinula edodes
Source: Sci Rep. 2015 Jun 9;5:10047. doi: 10.1038/srep10047 (PMC4460571; doi:10.1038/srep10047)
Supplement: Supplementary Information [file srep10047-s1.doc]

Supplementary Information

A novel cysteine desulfurase influencing organosulfur compounds in *Lentinula edodes*

Ying Liu^1,2^, Xiao-Yu Lei^1^, Lian-Fu Chen^2^, Yin-Bing Bian^2^, Hong Yang^1^, Salam A. Ibrahim^3^, Wen Huang^1,*^

^1^College of Food Science and Technology, Huazhong Agricultural University, Wuhan, Hubei, 430070, China, ^2^Institute of Applied Mycology, College of Plant Science and Technology, Huazhong Agricultural University, Wuhan, Hubei, 430070, China, ^3^Department of Family and Consumer Sciences, North Carolina A&T State University, 171 Carver Hall, Greensboro, NC 27411, United States.

^*^Corresponding author: Wen Huang, College of Food Science and Technology, Huazhong Agricultural University, No.1, Shizishan Street, Wuhan, Hubei, 430070, China

Telephone: 086-027-87282426

Fax: 086-027-87288373

E-mail address: huangwen@mail.hzau.edu.cn

Table S1 Kinetic constants for Lecsl (cysteine sulfoxide lyase and cysteine desulfurase activities)

| Substrate | *K*_m_ (mM) | *k*_cat_ (S^-1^) | *k*_cat_/*K*_m_ (M^-1^S^-1^) |
| --- | --- | --- | --- |
| S-methyl-L-cysteine sulfoxide  (cysteine sulfoxide lyase activity) | 26.17 ± 1.8 | 33.95 ± 1.5 | 1297 |
| L-cysteine  (cysteine desulfurase activity) | 1.72 ± 0.3 | 1.01 ± 0.2 | 586 |


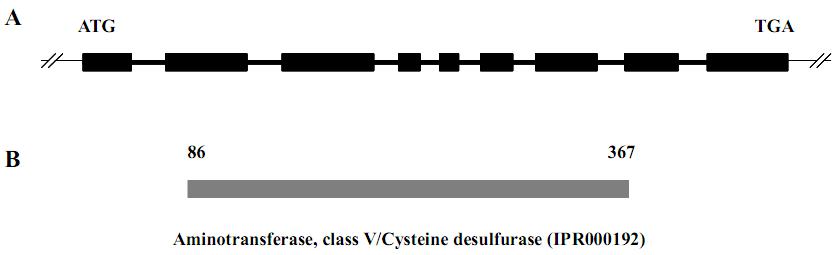


Figure S1 Gene and protein model of *L. edodes* cysteine sulfoxide lyase (Lecsl). (A) Structure of the putative Lecsl gene, exons are indicated by black boxed and introns are indicated by transverse line. (B) Protein model of Lecsl, conserved cysteine desulfurase domain is derived from InterProScan. The size and location of features are indicated by amino acid (AA) numbers.


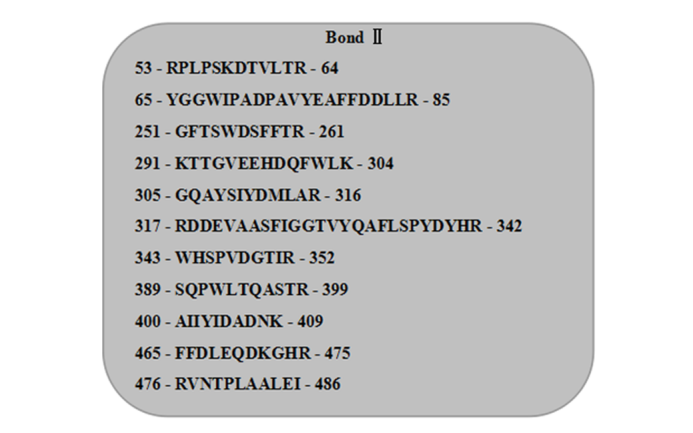


Figure S2 The protein band II was subjected to protein identification by MS/MS. Among the tryptic peptides obtained from the protein band, 11 peptides matched to a putative phosphatidylserine decarboxylase protein from *L. edodes*.


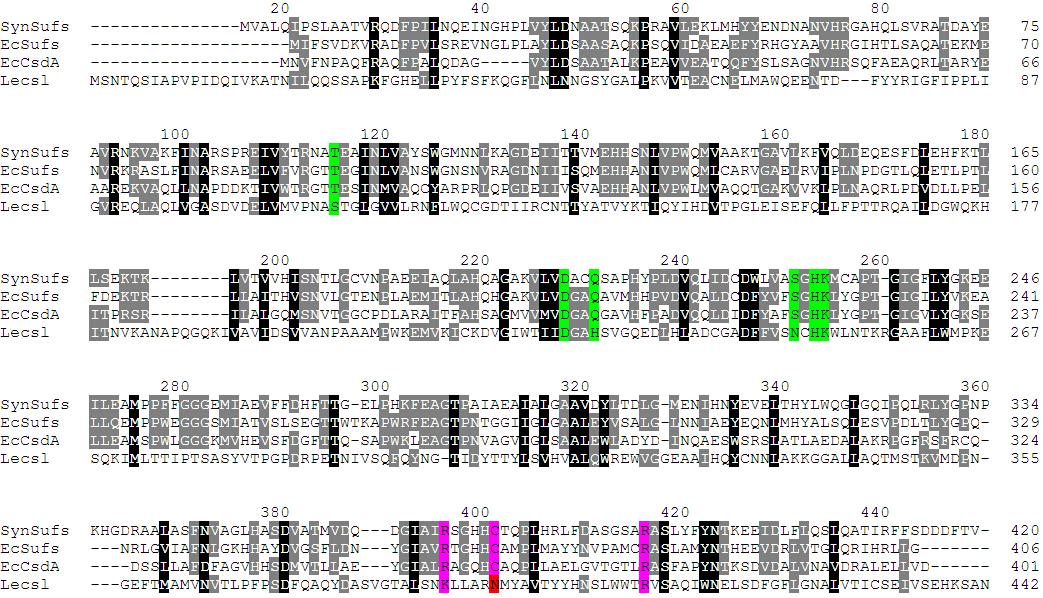


Figure S3 Multiple alignment of deduced amino acid sequences of Lecsl along with *Synechocystis sp*. (SynSufs, PDB: 1T3I), *Escherichia coli* (EcSufs, PDB: 1JF9) and *Escherichia coli* K-12 (EcCsdA, PDB: 4LW2). Conserved residues are shaded in black, similar residues in grey. Amino acid residues involved in substrate binding are highlighted in pink. Amino acid residues involved in PLP binding are highlighted in green. N393 of Lecsl is highlighted in red. The sequences were aligned with ClustalW, manually edited and visualized using GeneDoc.


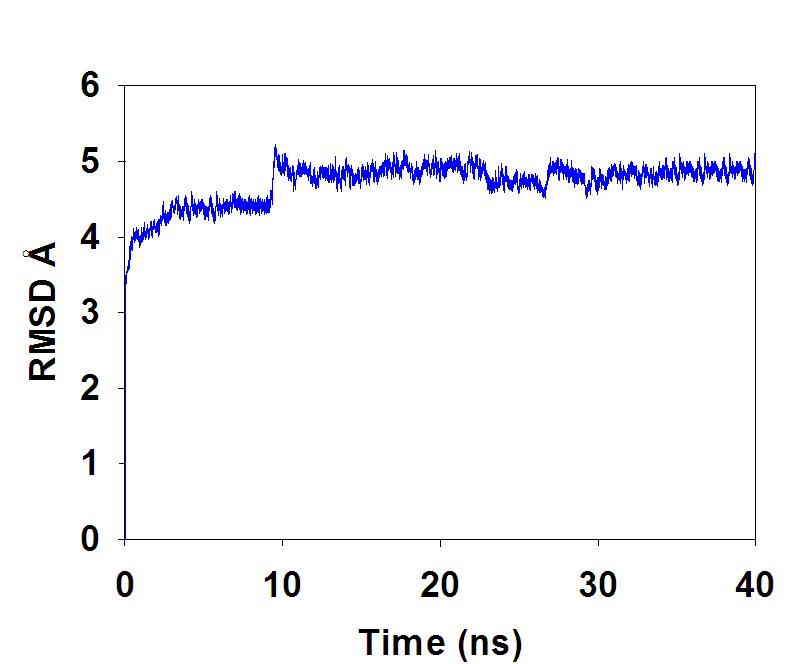


Figure S4 Plot of RMSD (in ångstrom) for the Lecsl-PLP complex during 40 ns MD

Simulation.


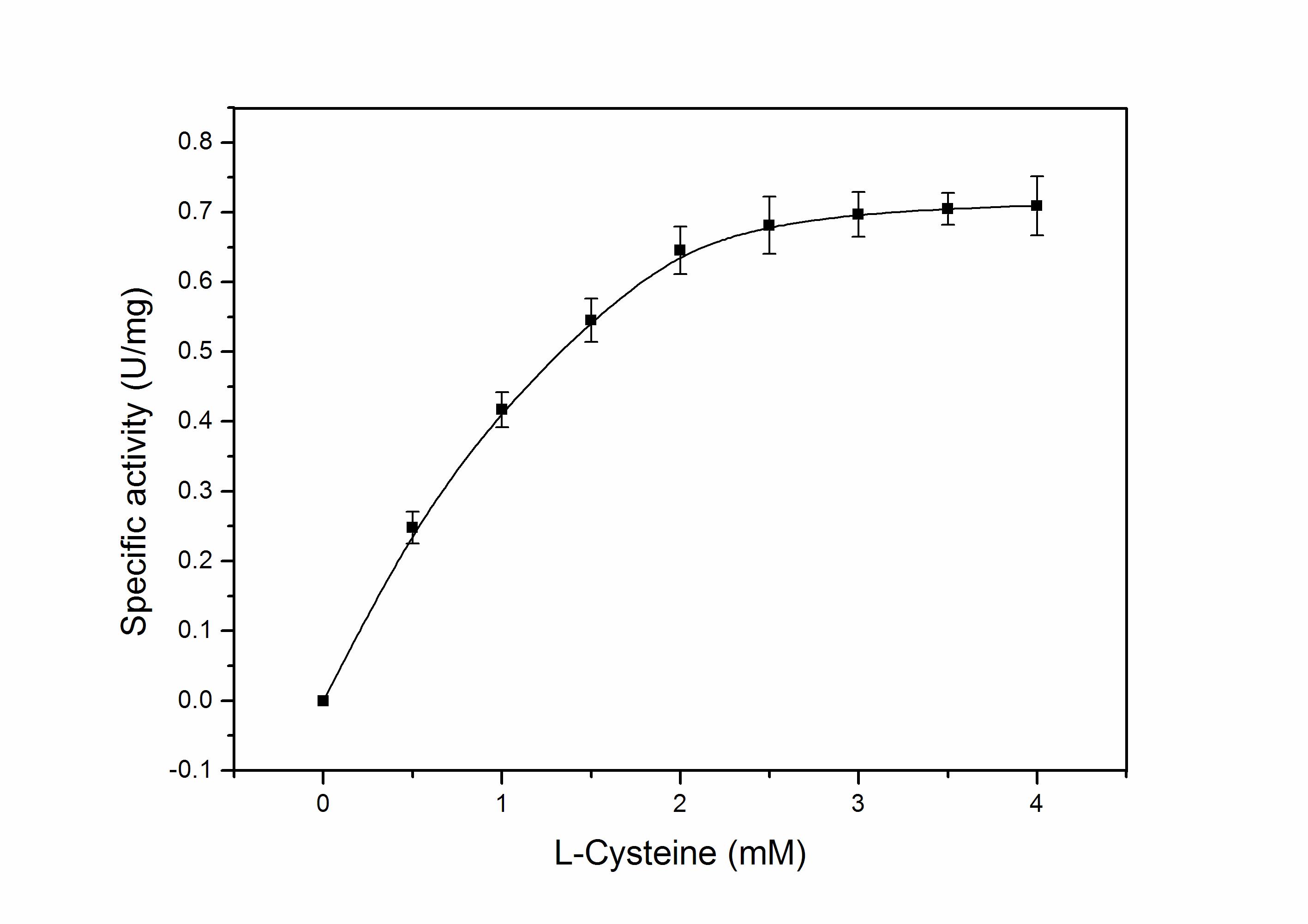


Figure S5 The cysteine sulfoxide lyase activity of Lecsl dependent on varied S-methyl-L-cysteine sulfoxide concentration.


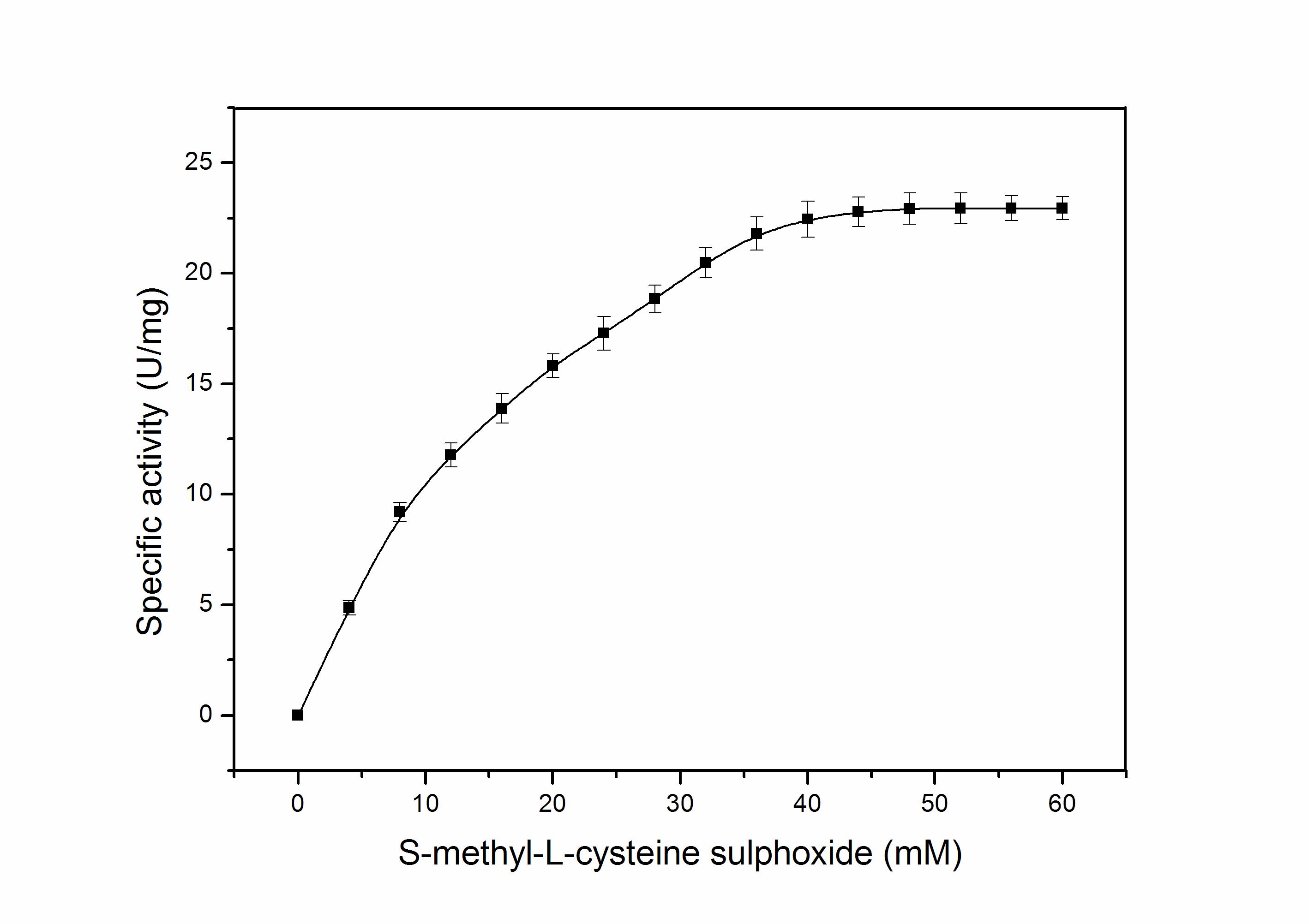


Figiure S6 The cysteine desulfurase activity of Lecsl dependent on varied L-cysteine concentration.
